# Skin Color Cues to Human Health: Carotenoids, Aerobic Fitness, and Body Fat

**DOI:** 10.3389/fpsyg.2020.00392

**Published:** 2020-03-11

**Authors:** David I. Perrett, Sean N. Talamas, Patrick Cairns, Audrey J. Henderson

**Affiliations:** School of Psychology and Neuroscience, Faculty of Science, University of St Andrews, St Andrews, United Kingdom

**Keywords:** health, skin color, carotenoids, fitness, body fat

## Abstract

Colorful carotenoid ornaments are sexually selected signals of health in many species. In humans too, carotenoids could provide a perceptible cue to health as they impart an attractive yellow-orange color to skin. Increasing carotenoid pigmentation and skin yellowness is associated with increased fruit and vegetable intake, but whether other aspects of human health benefit skin color is unknown. Carotenoids, as antioxidants, help maintain oxidative balance but are expended in this role. Therefore, any health factor affecting oxidative balance could alter the quantity of carotenoids available to color skin. Exercise increases endogenous antioxidant capacity and consequently may decrease expenditure of carotenoids. Fitness could also raise skin carotenoids by lowering body fat (a source of oxidative stress). Here we investigate the relationship between skin color (measured spectrophotometrically), aerobic fitness (measured by estimating the maximum volume of oxygen that a person can use per unit of time, VO_2_ max), and body fat. In a cross-sectional design, we find that both higher aerobic fitness and lower body fat are predictors of skin yellowness, independent of each other and dietary fruit and vegetable intake. In a longitudinal design over 8 weeks, we found that increase in fitness and decrease in body fat were independently associated with an increase in skin yellowness. Change in self-reported stress and sleep were further predictors of skin yellowness indicating a more general relation between health and skin tone. Simulations of the skin color associated with higher fitness were found to appear healthier. Hence, our results suggest that increasing cardiovascular fitness and decreasing fat levels produce a healthier skin color. Such findings have repercussions for public health because improved attractiveness can provide an incentive for a healthier lifestyle, including exercise and weight regulation.

## Introduction

In many species of birds and fish, yellow-red carotenoid pigments are prevalent as ornamentation in beaks, feathers, skin, and scales (Goodwin, 1986). Such ornaments are sexually selected; for example, in guppies (*Poecilia reticulata*) females preferentially mate with the most brightly colored males (Kodric-Brown, 1985), and in house finches (*Carpodacus mexicanas*) males with naturally or artificially brighter carotenoid plumage are more likely to mate (Hill, 1991). Carotenoid ornaments are thought to be sexually selected because they represent an honest signal of health (von Schantz et al., 1999). Evidence from a variety of species supports the idea of health signaling. For example, an increased parasite load decreases the brightness of orange spots in male guppies (Houde and Torio, 1992) and red coloration of male sticklebacks (*Gasterosteus aculeatus*) (Milinski and Bakker, 1990). In male greenfinches (*Carduelis chloris*), the size of the carotenoid plumage area is correlated with the ability to resist viral infection (Lindström and Lundström, 2000).

Carotenoids may play a role in human health and interpersonal attraction. Carotenoids occur naturally in plants but cannot be synthesized by animals. Hence, humans like other animal species obtain carotenoids through their diet. Once ingested, blood-borne carotenoids are secreted onto the skin where they impart a yellow-orange hue (Edwards and Duntley, 1939; Stamatas et al., 2004; Coetzee and Perrett, 2014) which is perceived as healthy and attractive (Stephen et al., 2011; Lefevre and Perrett, 2015). Skin carotenoid levels therefore reflect a good diet—rich in fruit and vegetables (Stephen et al., 2011; Whitehead et al., 2012)—but carotenoids could also act as a more general cue to human health. Reduced blood plasma carotenoid levels have been linked to a variety of disease states including HIV infection (Friis et al., 2001), malaria (Das et al., 1996), risk of some cancers (Leoncini et al., 2015; Huang et al., 2016), and myocardial infarction (Street et al., 1994); however, objective measures of health in terms of immune function, sperm status, and oxidative stress in normal adults have not been found to relate consistently to skin carotenoids (Foo et al., 2017a, b; Phalane et al., 2017). Our study explores the relationship between other aspects of health and carotenoid-based skin color.

High carotenoid levels in fish and bird ornaments may signal health by indicating capability to access dietary resources (Hill, 2000) or the ability to resist oxidative stress. As antioxidants, carotenoids can neutralize reactive oxygen species which contribute to oxidative stress, faster aging, and a variety of chronic diseases (Dowling and Simmons, 2009). This role of carotenoids prevents oxidative damage to lipids, proteins, and DNA, but carotenoids are irreversibly destroyed in the buffering process. There may therefore be a trade-off between carotenoid display in ornamentation, and their physiological utilization with only the healthiest individuals able to display, rather than utilize, their carotenoid reserves (von Schantz et al., 1999). Carotenoid pigmentation could also provide a cue to healthy levels of other non-colorful antioxidants (Pike et al., 2007; Vinkler and Albrecht, 2009).

Carotenoids are commonly discussed as indicators of resistance to disease but they may provide a cue to other aspects of health. There is consensus that exercise benefits health and conversely inactivity predisposes a host of unhealthy conditions including insulin resistance and raised blood levels of low density lipoproteins (Warburton et al., 2006; Janssen and LeBlanc, 2010). Different forms of exercise benefit health in different ways; endurance training raises aerobic fitness while resistance training increases strength. Carotenoid levels could act as a visible cue to general health through a relation to fitness.

Increased participation in exercise increases the body’s burden of oxidative stress (Jackson, 2000). Adaptations induced by exercise include an increased endogenous antioxidant capacity (Ristow et al., 2009). Specifically, endurance exercise training upregulates the activity of superoxide dismutase and glutathione peroxidase and increases total glutathione (Powers et al., 1999). Together these endogenous antioxidants not only have utility in sport (e.g., by delaying muscle fatigue) but they may also confer health benefits by reducing baseline oxidative stress (Powers et al., 1999). Fitter individuals maintain greater antioxidant capacity, despite enduring bouts of increased levels of pro-oxidants during exercise (Kriketos et al., 2000). It is therefore anticipated that increased fitness will increase carotenoid skin pigmentation if carotenoid pigmentation is indicative of antioxidant reserves (Vinkler and Albrecht, 2009). In essence the training required for fitness upregulates the production of the body’s own antioxidants, and this means that carotenoids are spared from expenditure and can accumulate in the skin. Fitness should therefore be associated with raised skin yellowness.

Fitness could also be related to carotenoid levels through a variety of indirect routes (including body fat, illness, psychological stress, and sleep quality). The most likely of these is through the association of fitness with low levels of body fat (Kriketos et al., 2000; Mota et al., 2002). High fat levels in unfit individuals could assimilate fat-soluble carotenoids (Chung et al., 2009) or increase oxidative stress (Vincent et al., 2007) thereby expending carotenoids. Indeed, high levels of body fat are associated with reduced carotenoid levels in plasma (Burrows et al., 2015) but the effects of body fat on carotenoid-based skin color are unknown. Hence, we explore body fat for its effect on skin color and its potential role in explaining any association between fitness and on skin color.

In study 1, we examine the effects of current aerobic fitness (measured using VO_2_ max) on carotenoid pigmentation (estimated by reflectance spectroscopy) between participants. Our main hypothesis is that higher participant fitness will relate to greater skin yellowness indicative of carotenoid pigmentation. We also anticipate a negative impact of body fat on skin yellowness. Body fat levels, infection rates, psychological stress, sleep, and dietary fruit and vegetable intake may each affect carotenoid levels and may provide pathways through which fitness could be associated with carotenoid levels and skin color. We therefore explore the influence of these variables on skin color using multiple regression.

Our study explores the links between aspects of health and skin carotenoids. We do not investigate the antecedents of health, yet the antecedents are worth consideration as they have consequences for the interrelationship between health variables measured.

The Personality Inventory for the Diagnostic and Statistical Manual of Mental Disorders-Fifth Edition reveals five factors of maladaptive aspects of personality (antagonism, detachment, negative affectivity, disinhibition, and psychoticism). Individually and collectively these personality traits are associated with health risk factors including high body weight, unhealthy diet and insufficient physical activity (Góngora and Castro Solano, 2017). Twin studies indicate that maladaptive personality traits have a heritability of 30–50% (Wright et al., 2017). Hence, there are clear genetic predispositions to poor diet, low exercise, and high body fat.

It is also clear that there are psychosocial determinants of health with a disadvantaged upbringing leading to poor health and health behavior in adulthood (Bell, 2017). For example, residential area deprivation and lower socioeconomic status independently predict decreased fruit and vegetable consumption (Shohaimi et al., 2004; Lakshman et al., 2010). Poor and disadvantaged groups are more likely than their better-off counterparts to be exposed to stressors and these exact a toll on health (Braveman, 2014). Socially disadvantaged groups are five times more likely than advantaged groups to engage in unhealthy behavior, including eating a poor diet and taking too little physical exercise (Buck and Frosini, 2012). Indeed, several studies report that increased sedentary behavior and decreased physical activity are related to background deprivation (Hillsdon et al., 2008; Amuzu et al., 2009; Stamatakis et al., 2009).

The link between social background and mental and physical health appears to be mediated by a sense of personal relative deprivation, i.e., negative emotions elicited when comparing self to others, e.g., agreeing with the statement, “I feel resentful when I see how prosperous other people like me seem to be” (Callan et al., 2015). Those scoring high on personal relative deprivation are likely to report lower levels of physical and mental health, greater levels of self-reported stress, and lower sleep quality (Callan et al., 2015).

Both personality and psychosocial influences on health predict a positive relation between health measures. Individuals with high fitness are more likely to have low body fat and to report high fruit and vegetable consumption, good sleep, and low levels of sickness and stress.

## Study 1

In this study, we compared the skin color of participants with different levels of fitness and body fat in a cross-sectional design.

### Methods

Methods follow our pre-registration at osf.io/ay2kq unless otherwise noted and were approved by St Andrews University Teaching and Research Ethics Committee (project number PS12005). Participants gave informed consent and were reimbursed at a rate of £5 per hour. Procedures were carried out in accordance with the relevant guidelines and regulations. Informed consent was obtained from all subjects.

#### Research Participants

We recruited 180 participants who completed the study over two weekends. After exclusions (see below), a total of 134 participants remained (71 women, 63 men, *M*_age_ = 21.32, *SD* = 2.38, range: 18–32). Ethnicity of our sample (White = 81%, Indian = 3%, Pakistani = 2%, Chinese = 8%, South East Asian < 1%, Other Asian 2%, Hispanic < 1%, Arab < 1%, mixed or multiple ethnic groups = 4%) reflected that of the University of St Andrews student population. Recruitment involved poster and online Facebook advertising, weekly memos to staff, students, and emails to all sports clubs.

##### Skin color

Skin color was measured using a spectrophotometer (Konica Minolta CM-700d, d65 illuminant, 8° illumination angle, specular component excluded). The spectrophotometer recorded the light reflected from the skin at 10 nm intervals from 400 to 700 nm and provided numerical color values in CIE L^∗^ a^∗^ b^∗^ color space. L^∗^ represents darkness/lightness on a scale from 0 (black) to 100 (white); a^∗^ represents a scale from green (negative values) to red (positive values); and b^∗^ represents a scale from blue (negative values) to yellow (positive values). In the context of skin color, only positive values are relevant and so a^∗^ and b^∗^ are referred to as “redness” and “yellowness,” respectively, and L^∗^ will be referred to as “lightness” (Stamatas et al., 2004). Skin color was measured at six body locations (palm, inner forearm, triceps, shoulder, cheek, and forehead); three measures were taken at each location. For each color channel (lightness, redness, and yellowness), data were averaged across three measurement repetitions and six locations for each participant to produce global color values. The range of color values across the sample was L^∗^ (*M* = 64.65, *SD* = 2.88, range: 56.02–69.87), a^∗^ (*M* = 9.36, *SD* = 1.33, range: 5.96–12.62), and b^∗^ (*M* = 16.64, *SD* = 2.04, range: 12.22–22.28).

#### Body Composition

After measuring each participant’s height, foot to foot bioelectrical impedance was measured using the TANITA SC-330 scale. Measurements of weight and impedance were combined with participant’s gender, height, body type (athletic: > 10 h vigorous exercise a week, normal: < 10 h a week), and age (manually input to the device) to determine body fat% (Jebb et al., 2000). Body fat varied across gender, with women (*M* = 25.20, *SD* = 6.45, range: 11.9–41.5) having higher average fat percentage than men (*M* = 11.94, *SD* = 4.92, range: 3.7–29.1).

#### Diet

A validated eight-item fruit and vegetable servings per day questionnaire (Bogers et al., 2004) was used to assess participants’ typical daily intake (*M* = 3.67, *SD* = 2.31, range: 0.05–9.66) as an indication of carotenoid intake.

#### Illness

To assess the physical illness of participants, the Symptoms of Illness Checklist (*SIC*, *M* = 6.87, *SD* = 6.72, range: 0–29) was used (Stowell et al., 2009). This questionnaire was shortened from the original to include 12 symptoms related to colds and flu (i.e., sore throat, coughing, feeling exhausted or fatigued, lightheaded, faint or dizzy, muscle aches or pain not due to strenuous exercise, sinus problems, nasal problems, nausea, headaches, fever, change in appetite, swollen glands in neck). The questionnaire asked participants to report the frequency with which they experienced these symptoms (i.e., 1–3, 4–7, 8–14, 15–49, or 50–60 days) over the past 2 months and the impact that these symptoms had on their daily activities (i.e., no interference with daily activities, some interference, interference with daily activities, or severe interference). Total score = sum (frequency ^∗^ severity for each symptom).

#### Stress

Recent psychological stress (*M* = 5.06, *SD* = 4.03, range: 0–17) was assessed using the seven-item stress subscale of the Depression, Anxiety, and Stress Scales (DASS–21) (Henry and Crawford, 2005).

#### Sleep

Typical sleep patterns (*M* = 7.41, *SD* = 0.79, range: 5–10 h) were assessed by asking participants “on average, how many hours of sleep do you get in a 24 h period (to the nearest half hour)?”

#### Aerobic Fitness

A modified version of the Bruce submaximal treadmill Graded Exercise Test (Hill and Timmis, 2002) was used to assess VO_2_ max expressed relative to a person’s weight (*M* = 46.41, SD = 7.42, range: 34.5–69.3 ml/kg/min). Participants started at a slow walk of 1.7 mph at an incline grade of 10%. After the first 3 min, the incline was increased to 12% and speed to 2.5 mph. After another 3 min, the incline was increased to 14% and speed to 3.4 mph. After a final 3 min, participants cooled down for 1 min at an incline of 0% and a speed of 1.7 mph. At each minute, participants were asked about their rate of perceived exertion (RPE, on a scale from 6 to 20). The test was terminated if the participant asked to stop (one participant) or if the RPE reached 18 (one participant). Heart rate (beat-to-beat interval) was recorded during the test and analyzed using SPORTS software (version 4.7.1.2, Firstbeat Technologies Ltd., Jyväskyla, Finland, with an artifact detection filter). Age-predicted maximum heart rate (HR max) was calculated for each participant as 210 – (0.65 × age). The treadmill test protocol was entered into Firstbeat SPORTS software and VO_2_ max was predicted from the linear relation between workload and heart rate extrapolated to the age-predicted HR max. Maximal workload was then converted to theoretical VO_2_ max (Hill and Timmis, 2002).

#### Procedure

On arrival, participants were asked to wipe their face with a hypoallergenic wipe in preparation for skin color measurements. After skin color measurements, participants were seated in a quiet, private setting for questionnaires. Participants then lay down for 5 min for additional heart rate measures. Lastly, approximately 30 min after registration, participants completed the treadmill aerobic fitness test.

#### Statistical Analysis

To understand the interrelationships among independent and dependent variables, we report partial correlations controlling for participant gender. For more definitive analysis, we use multiple regression to investigate the effects of fitness and body fat on skin yellowness. The model included control variables, gender and skin lightness (L^∗^), and covariates, fruit and vegetable consumption, illness, stress, sleep, and supplements. Regression residuals were normally distributed. Regression excluding data points with high influence (high Cook’s distance) did not materially change the pattern of results.

#### Exclusions

Exclusions included participants who self-reported wearing fake tan (*n* = 4) and participants whose heart rate monitoring reported a measurement error above 35% (*n* = 20; cut-off criteria recommended by Firstbeat). Alcohol the night before can reduce aerobic fitness by 11% (O’Brien, 1993), therefore two participants reporting > six drinks of alcohol the prior day were excluded. Forty-six (33%) participants reported taking dietary supplements (with antioxidant properties). We therefore included supplements (yes/no) in analysis, rather than excluding a third of the sample.

#### Outliers

As pre-registered, we checked all variables for normality and outliers that were > 3SD from the mean were excluded. Outliers excluded by this criterion included five in age, three in fruit and vegetable intake, five in illness, one in sleep, one in stress, one in VO_2_ max, and four in skin lightness. Skew and kurtosis were acceptable after natural logarithm transforms of VO_2_ max and square root transform of illness, and stress variables.

#### Control Variables

Both carotenoid pigments and melanin increase skin yellowness but melanin additionally decreases skin lightness (L^∗^) whereas carotenoids have little effect on L^∗^ (Alaluf et al., 2002; Stamatas et al., 2004). Accordingly, L^∗^ is entered into the regression model assessing the relation between fitness and skin yellowness. This control means that any relationship between b^∗^ and fitness cannot be attributed to melanin. Gender influences human skin reflectance. Women of all ethnicities are lighter on average than men (Jablonski and Chaplin, 2000). Gender is also related to VO_2_ max with men generally having higher values than women (Berndtsson et al., 2007). Hence, gender is controlled for in our analysis. Gender is coded as women 0, men 1.

#### Spectral Analysis

Multiple regression (used above to investigate skin yellowness) was applied to investigate the impact of fitness on skin reflectance at 10 nm intervals from 400 to 600 nm (wavelengths associated with light absorption by carotenoids and hemoglobin). While controlling for other variables, this regression was used to generate standardized β coefficient values for the relationship between fitness and reflectance every 10 nm. The spectral tuning of the standardized coefficient values was then compared to the absorption spectra of individual pigments (Stephen et al., 2011; Whitehead et al., 2012; Phalane et al., 2017).

### Results

Supporting the main hypotheses, partial correlations in Table 1 show that, while controlling for gender, skin yellowness relates both positively to fitness and negatively to body fat%. Fitness and body fat are also negatively related; hence, Table 1 does not separate the associations between skin color, fitness, and fat. Moreover, increased fruit and vegetable consumption shows a trend to relate to skin yellowness and to fitness. The regression analysis below resolves some of the ambiguity in these statistical relationships.

**TABLE 1:** Partial correlations between predictors of skin color controlling for gender.

	L*	a*	b*	VO_2_	Fat	F and V	Stress	*SIC*	Sleep
a* (red)	−0.71**								
b* (yellow)	−0.63**	0.37**							
VO_2_ max	0.08	0.10	0.17*						
Fat%	0.02	–0.09	−0.19*	−0.29**					
Fruit and veg.	–0.07	0.09	0.16^†^	0.15^†^	0.02				
Stress	0.04	–0.01	–0.06	0.06	–0.05	0.06			
*SIC* (illness)	0.08	0.04	–0.07	–0.13	0.03	−0.16^†^	0.16^†^		
Sleep	0.07	–0.03	–0.01	0.07	–0.07	–0.03	–0.04	0.07	
Supplements	–0.03	–0.08	0.06	0.02	–0.01	–0.02	0.21*	0.14^†^	0.26**

Skin lightness (L*), redness (a*), and yellowness (b*). Statistically significant relationships are in bold (**P < 0.005, *P < 0.05, ^†^*P* < 0.1, two-tailed probabilities). Supplements are coded none = 0, taking supplements = 1. F and V = Fruit and vegetables.

#### Skin Yellowness

**FIGURE 1:**
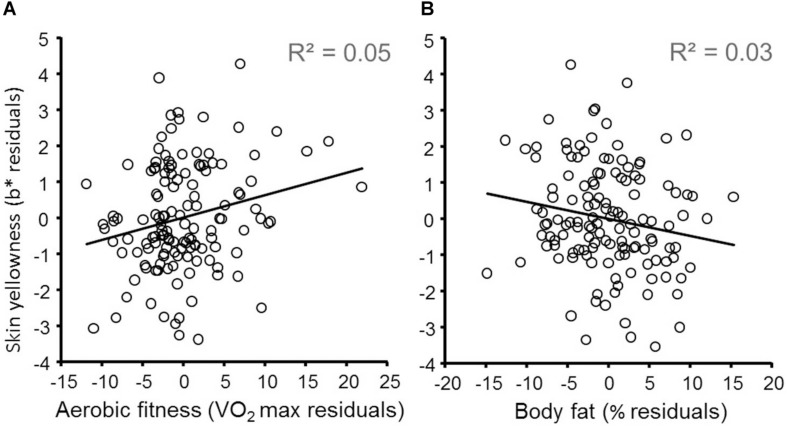
Effects of aerobic fitness and body fat on skin yellowness. **(A)** Variation in skin yellowness (not explained by gender, skin lightness, fruit and vegetable consumption, supplements, and body fat) is plotted against variation in fitness (controlling for the same variables). The diagonal line denotes the best fit regression (*N* = 134). Note that while a natural log transformed VO_2_ max was used in the regression model, for illustration VO_2_ max was not log transformed for more meaningful visualization. **(B)** Variation in skin yellowness (not explained by gender, skin lightness, fruit and vegetable consumption, supplements, and fitness) is plotted against variation in body fat% (controlling for the same variables).

The results of linear regression predicting participants’ skin yellowness are given in Table 2 and indicate that both fitness and body fat independently predict skin yellowness. Skin lightness, gender, VO_2_ max, and body fat% are all independent significant predictors of skin yellowness [overall model: *R*^2^ = 0.49, *F*(9,124) = 13.18, *P* < 0.001]. Increasing aerobic fitness (Figure 1A) and reduced body fat% (Figure 1B) are associated with increased skin yellowness. As expected, higher skin yellowness is associated with darker skin (reduced L^∗^) attributable to higher melanin. Controlling for other variables, women have higher skin yellowness than men. Participant age could have been a confounding variable but adding age to the regression model did not change the relationship between skin yellowness, fitness, and body fat. Moreover, in the extended regression model, age was unrelated to skin yellowness (*P* = 0.785).

**TABLE 2:** Multiple regression with predictors of skin yellowness (b*).

Variable	*B*	*SE*	*CI*		β	*P*
			*Lower*	*Upper*		
Lightness	–0.47	0.05	–0.57	–0.37	–0.67	<0.01
Gender	–1.51	0.46	–2.43	–0.59	–0.37	<0.01
VO_2_ max	2.97	1.19	0.62	5.33	0.23	0.01
Body fat%	–0.05	0.02	–0.09	0.00	–0.21	<0.05
Fruit and veg. intake	0.08	0.05	–0.02	0.19	0.10	0.13
Stress	–0.30	0.26	–0.82	0.22	–0.08	0.25
*SIC* (illness)	0.04	0.10	–0.17	0.24	0.02	0.74
Sleep	–0.02	0.18	–0.37	0.33	–0.01	0.91
Supplements	0.24	0.29	–0.33	0.80	0.06	0.40

*Regression coefficients (*B*) and standard error (*SE*), confidence intervals (*CI*), standardized beta (β), and probability (*P*).*

#### Skin Redness

Although no hypotheses were made about other aspects of skin color, we used linear regression to explore the relation of fitness with skin lightness and redness. Neither VO_2_ max (*B* = 2.52, *SE* = 2.14, β = 0.14, *P* = 0.241) nor body fat (*B* = 0.03, *SE* = 0.04, β = –0.08, *P* = 0.553) were related to skin lightness [controlling other variables in Table 2, overall model: *R*^2^ = 0.163, *F*(8,133) = 3.046, *P* < 0.004], indicating that fitness and body fat are not related to level of melanin or suntan. Increased aerobic fitness (*B* = 1.65, *SE* = 0.64, β = 0.192, *P* = 0.011) but not fat level (*B* = –0.01, *SE* = 0.01, β = –0.04, *P* = 0.606) was related to increased skin redness [controlling Table 2 variables, overall model: *R*^2^ = 0.65, *F*(9,133) = 25.664, *P* < 0.001].

#### Spectral Analysis

**FIGURE 2:**
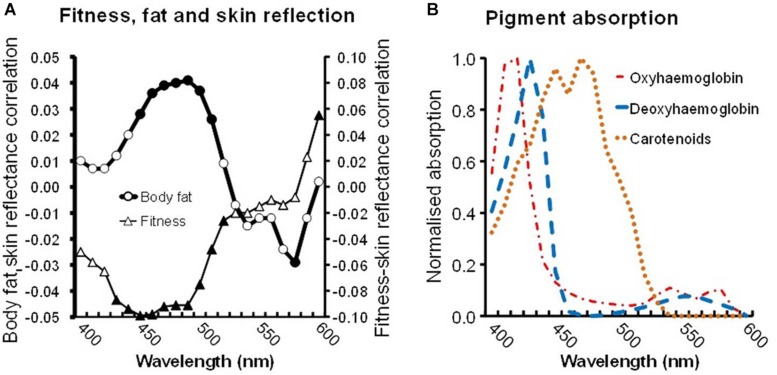
**(A)** Skin reflectance linked to fitness and to body fat. Triangles display the partial correlation between VO_2_ max and skin reflectance (controlling other variables, see text) at 10 nm intervals between 400 and 600 nm. Similarly, circles display the partial correlation between body fat and skin reflectance. Filled symbols denote significant correlations (*P* < 0.05). **(B)** Absorption spectra of skin pigments: oxyhemoglobin (red dashed dots), deoxyhemoglobin (blue dashes), and common carotenoids (average absorption of α, β-carotene and lycopene, orange dots). Note the fitness-reflectance and the fat-reflectance correlations are strongest for light of 450–500 nm, corresponding to the peak absorption of carotenoids.

The preceding results relate fitness (and decreased fat) to increased skin yellowness, yet yellowness could arise from blood pigments. While carotenoids are one explanation, a change in blood composition from less to more oxygenated hemoglobin might be involved. To investigate the pigments responsible, an analysis was performed of reflectance from the skin across the spectrum (Stephen et al., 2011; Whitehead et al., 2012; Phalane et al., 2017). Regression was used to generate standardized *b* coefficient values (equivalent to partial correlation values) for the relationship between fitness or body fat% and skin reflectance at 10 nm intervals from 400 to 600 nm (Figure 2A). This spectral tuning can then be compared to the absorption spectra of pigments (Figure 2B).

A negative relation between the fitness-reflectance tuning and carotenoid absorption spectra is expected, because high fitness should raise carotenoids, increasing the skin’s light absorption and producing a concurrent decrease in reflectance. We expect the relationship to be strongest (most negative) at wavelengths associated with the peak absorption by carotenoids (450–500 nm, Figure 2B), and weaker at other wavelengths (Stephen et al., 2011; Whitehead et al., 2012; Phalane et al., 2017).

Indeed at 430–520 nm wavelengths, the fitness relationship is strongest and significant (Figure 2A). Across a broader portion of the spectrum, 400–600 nm, the relationship between fitness and skin reflectance was significantly correlated with the mean absorption of three common carotenoids (β-carotene, α-carotene, and lycopene, Spearman’s ρ = –0.958, *N* = 21, *P* < 0.0005) but was not correlated with the absorption spectrum of oxyhemoglobin or deoxyhemoglobin (ρ = –0.327, *P* = 0.148; ρ = –0.064, *P* = 0.782).

Note that for body fat, the relation to reflectance is the reverse of that for fitness. High fat levels presumably mean a paucity of carotenoids, decreasing the light absorption and producing increasing reflectance. Again the fat-reflectance relationship is strongest (most positive) and significant at 450–510 nm (Figure 2A circles), the peak absorption by carotenoids. Across the 400–600 nm range, the relationship between body fat and skin reflectance was significantly correlated with carotenoid (ρ = 0.896, *P* < 0.0005) but not with the oxyhemoglobin or deoxyhemoglobin absorption spectra (ρ = –0.114, *P* = 0.622; ρ = –0.320, *P* = 0.157).

### Discussion

Study 1 found that two objective measures of health (aerobic fitness and body fat%) in normal adults are related to variation in skin color across individuals. Difference in skin tone across individuals is usually thought of in terms of melanin. We found evidence that health-related skin color variation is attributable to carotenoid pigments (which affect skin yellowness) and is unrelated to melanin (which affects skin lightness). The manner in which fitness and fat level varied with light reflected from the skin across the spectrum was found to parallel carotenoid absorption spectra (Stephen et al., 2011; Whitehead et al., 2012) (Figure 2), further implicating skin carotenoids as the responsible pigments.

Aerobic fitness was found to predict skin yellowness, independent of other potentially confounding variables (gender, body fat%, diet, stress, illness, sleep, supplements, and melanin levels). While body fitness and fat levels were correlated, regression analysis showed that their influence on skin yellowness was largely independent.

## Study 2: Change in Fitness and Body Fat

### Introduction

Study 1 compared people at different levels of fitness and body fat. Study 2 compared changes in skin color with changes in fitness and body fat in a within-participant design. As for study 1, we also controlled for other variables that might affect skin color. Here we controlled for changes in dietary intake of fruit and vegetables, changes in dietary supplements, changes in patterns of sleep, changes in stress, and changes in skin lightness (L^∗^) which might reflect a change in melanin level. From study 1, we hypothesized that an increase in aerobic fitness would relate to an increase in skin yellowness, whereas an increase in body fat% would be accompanied by a decrease in skin yellowness.

### Methods

#### Ethics Statement

The study received ethical approval from the School of Psychology and Neuroscience Ethics Committee (approval code PS11146). Prior informed written consent was obtained from all participants. All participants were reimbursed for their time at a rate of £5 per hour.

#### Participants

Recruitment took place primarily from within university sports teams at the University of St Andrews, as their physical training during the term was anticipated to change fitness level within participants. Students participated in two sessions held in early September and late November. After exclusions a total of 59 participants remained (26 women, 33 men, *M*_age_ = 21.08, *SD* = 2.39, range: 17–30). The ethnicity of the sample (White = 88%, Chinese = 7%, Other Asian 2%, mixed or other ethnic groups = 3%) reflected that of the University of St Andrews student population.

#### Procedure

The procedure was similar to study 1 except for the omission of the treadmill fitness test and inclusion of Polar watch estimate of VO_2_ max from heart rate variability (Polar Electro, 2016). Participants were tested at a similar time of day on two occasions (after the summer vacation and 8 weeks later). For the Polar fitness test, gender, age, height, body weight, and self-assessed physical activity (0–1, 1–3, 3–5, 5–8, 8–12 h per week) were entered into the watch. A heart rate monitor with electrode gel was fastened around the chest at the level of the sternum. Participants were tested lying down with hands by their sides in a peaceful, dimly lit, and quiet environment. Participants relaxed prior to the test which took 5 min. Questionnaires requested self-report of diet, supplement use, stress over the past week, and usual amount of sleep. Report of sickness was requested over the last 8 weeks.

#### Statistical Analysis

For each variable, the change from session 1 to 2 was computed. As with study 1 we used multiple regression to investigate the effects of change in fitness and body fat on change in skin yellowness. Change in skin yellowness was the dependent variable and changes in fitness and body fat were predictors. The model included control variables, namely, changes in skin lightness (L^∗^), fruit and vegetable consumption, antioxidant dietary supplements, illness, stress, and sleep. Probabilities quoted are two-tailed except for those associated with the directional hypotheses for change in fitness and change in body fat. Regression analysis with no exclusions revealed that increase in aerobic fitness, increase in fruit and vegetable intake, increase in usual sleep, decrease in skin lightness, and decrease in stress were all significant predictors of increased skin yellowness (all *P* < 0.024, two-tailed probability).

#### Exclusions and Outliers

Fitness testing, skin color measures, and questionnaires were administered on two sessions for 69 participants. Two participants were excluded for reporting fake tan in session 1 and one with low skin lightness. Outliers > 3SD from the mean of the change in values between sessions 1 and 2 were excluded. These included one in skin lightness change, one in fat change, two in aerobic fitness change, one in illness change, and one with illness data missing for one session, one usual sleep duration change, and one in fruit and vegetable intake change. Skew and kurtosis across all change variables were acceptable.

### Results

Table 3 presents Pearson’s correlations between variables of interest. The table does not reveal any relationship between change in fitness or body fat% and skin color change. Instead, the table reveals that a decrease in stress and an increase in fruit and vegetable intake are associated with an increase in skin yellowness. Associations between change in skin color and changes in fitness or body fat% could be masked by these relationships or confounded by changes in skin lightness and other variables. The regression analysis below explores the relationship between each predictor variable and skin yellowness controlling for all other variables and hence provides a more definitive picture.

**TABLE 3:** Correlations between predictors of change in skin color.

	L* change	a* change	b* change	VO_2_ change	Fat change	F and V change	Stress change	*SIC* change	Sleep change
a* (red) change	−0.57**								
b* (yellow) change	–0.16	0.07							
VO_2_ max change	0.19	–0.23	0.06						
Fat% change	0.07	0.01	–0.21	0.06					
Fruit and veg. change	–0.09	0.02	0.30*	0.00	0.13				
Stress change	0.09	0.02	−0.29*	0.20	0.20	–0.19			
*SIC* (illness) change	0.21	–0.16	–0.11	–0.03	−0.33*	–0.17	–0.12		
Sleep change	–0.03	–0.02	0.18	0.01	0.12	–0.03	0.34*	0.12	
Supplements change	0.21	–0.12	–0.01	0.04	–0.01	0.02	0.16	0.21	0.30*

Skin lightness (L*), redness (a*), and yellowness (b*). Statistically significant relationships are in bold (**P < 0.005, *P < 0.05, two-tailed probabilities). Supplements are coded as stopped taking supplements = −1, no change = 0, started taking supplements = 1, F and V = Fruit and vegetables.

**FIGURE 3:**
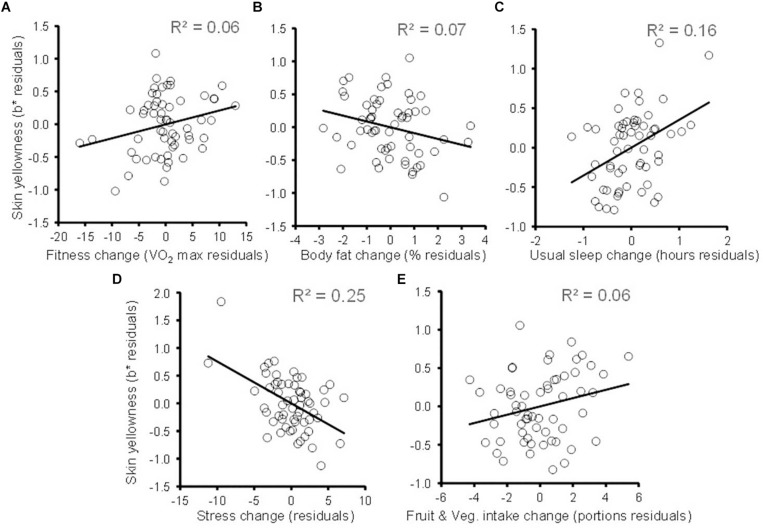
Changes in skin yellowness associated with change in health-related variables. Partial plots following conventions of Figure 1. **(A)** Skin yellowness change (not explained by changes in skin lightness, body fat, sleep, stress, fruit and vegetable intake, illness, and dietary supplements) is plotted against change in fitness (controlling for the same variables). The diagonal line denotes the best fit regression. Similar partial plots relating change in skin yellowness to changes in **(B)** body fat%, **(C)** habitual sleep, **(D)** self-reported stress, and **(E)** fruit and vegetable intake. Each partial plot controls for the other variables in the regression model.

The results of linear regression predicting change in participants’ skin yellowness are given in Table 4 and indicate that both change in fitness and change in body fat independently predict change in skin yellowness (both *P* ≤ 0.04, one-tailed probability) [overall model: *R*^2^ = 0.423, *F*(8,58) = 4.574, *P* < 0.001]. Increasing aerobic fitness (Figure 3A) and reduced body fat% (Figure 3B) are associated with increased skin yellowness. Additional control measures were associated with skin color change. A reduction in the duration of usual sleep (Figure 3C) and an increase in stress (Figure 3D) were associated with reduced skin yellowness. Increased fruit and vegetable intake showed a trend to be associated with increase in skin yellowness (Figure 3E). Analysis of influence and leverage in the regression model revealed five data points to have high influence (Cook’s distance > 4/sample size). Exclusion of these data did not change the pattern of significant variables except that the fruit and vegetable intake became a significant predictor (*P* = 0.002). Age could have moderated skin color change but adding age to the regression model did not change the pattern of results and age was itself unrelated to yellowness change (*P* = 0.447).

**TABLE 4:** Multiple regression with predictors of change in skin yellowness (b*).

Variable	*B*	*SE*	*CI*		β	*P*
			*Lower*	*Upper*		
Lightness change	–0.09	0.08	–0.25	0.07	–0.13	0.27
VO_2_ max change	0.02	0.01	0.00	0.04	0.20	0.04*
Body fat% change	–0.09	0.05	–0.18	0.01	–0.22	0.03*
Fruit and veg. intake change	0.05	0.03	–0.01	0.11	0.20	0.08
Stress change	–0.08	0.02	–0.11	–0.04	–0.48	<0.01
Sleep change	0.35	0.11	0.12	0.58	0.38	<0.01
*SIC* (illness) change	–0.01	0.00	–0.01	0.00	–0.15	0.23
Supplement change	0.03	0.16	–0.30	0.36	0.02	0.87

Regression coefficients (*B*) and standard error (*SE*), confidence intervals (*CI*), standardized beta (β), and probability (*P*). *One-tailed probability.

Multiple regression predicting skin lightness change revealed that none of the change variables in Table 4 were related to change in skin lightness [all *P* > 0.11, overall model: *R*^2^ = 0.158, *F*(7,51) = 1.37, *P* = 0.238]. This finding indicates that changes in fitness, body fat, stress, sleep, and fruit and vegetable intake are unrelated to level of melanin or suntan. Similarly, regression showed no associations between change in skin redness and change in any of health-related variables in Table 4 [all *P* > 0.32; overall model: *R*^2^ = 0.38, *F*(8,50) = 3.80, *P* < 0.001, although skin redness change was predicted by skin lightness change *P* < 0.001].

### Discussion

Study 1 showed that fitness and fat level were independently associated with skin yellowness in a cross-sectional survey. Study 2 extends these findings by tracking changes in individuals in a longitudinal design. Increasing fitness and decreasing fat levels over an 8 week period were independently associated with a rise in skin yellowness.

Regression showed multiple independent associations between changes in skin color and changes in variables affecting health. A reduction in skin yellowness was independently related to a reduction in usual sleep, an increase in stress, and a trend to decreased fruit and vegetable intake. The association between change in skin yellowness and changes in fitness, body fat, sleep, stress, and fruit and vegetable intake all occurred while controlling for skin lightness. Indeed, changes in health variables were not related to change in skin lightness. As with study 1, study 2 the skin color—health associations appear to reflect carotenoid pigments in the skin rather than melanin, since melanin would reduce skin lightness.

In study 2, there was an interval of 8 weeks between measurements, hence the study suggests that skin color change in response to change in health status occurs within 8 weeks as has been seen in other studies (Whitehead et al., 2012; Coetzee and Perrett, 2014). Further study is required to define the minimum duration of changes to fitness, weight, stress, and sleep for pigment changes to be detectable.

## Study 3: Perception of Fitness-Associated Skin Color Changes

### Introduction

Studies 1 and 2 showed that increased fitness was associated with a change in skin color. Here we explore whether these fitness-induced changes in skin color are discernable by the human eye and whether they are perceived as healthy. Simulating the skin color change from extra fruit and vegetable consumption provokes an increased preference in interactive optimization and forced choice task (Stephen et al., 2011; Whitehead et al., 2012; Lefevre and Perrett, 2015). Here we test whether similar effects are evident for fitness associated skin coloration. We hypothesize that faces with high fitness related skin color is seen as healthier than equivalent faces with skin color adjusted to represent a color associated with lower fitness.

### Perceptual Methods

#### Participants

Twenty-one Caucasians (age: 25.7 ± 7.3 years, 14 women, seven men) participated in the perceptual study.

#### Stimuli

Two male and two female composite Caucasian faces (each comprised of three faces of the same sex blended together) were used as stimuli.

#### Image Transforms

**FIGURE 4:**
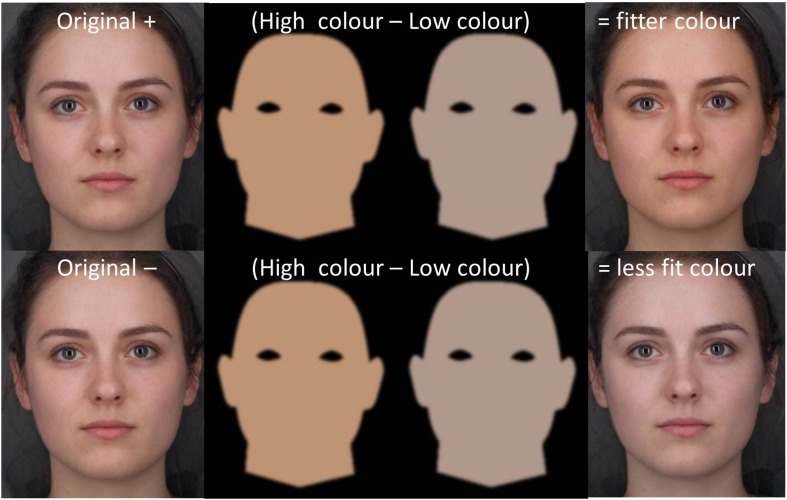
Visualization of the transformation used to alter skin color of stimuli. The “fitter” facial image was created by adding 0.25 of the difference between the High and Low color masks to the original facial image. Likewise, the “less fit” image was made by subtracting 0.25 of the difference. Facial images are averages of three individuals and do not represent a real identity. Written informed consent was obtained from all three individuals for the publication of their image.

The goal of transformation was to produce facial images varying in skin color associated with raised or lowered fitness (Stephen et al., 2011; Whitehead et al., 2012; Lefevre and Perrett, 2015). The mean level of fitness in the sample of study 1 was a VO_2_ max of approximately 50. We produced images with skin color simulating a range in VO_2_ max from 10 to 90 (see Figure 4 right). To achieve this, two facial masks with even skin color were created in MATLAB (Figure 4 middle pair). The “High” color represents skin color associated with high fitness and the “Low” color represents skin color associated with low fitness. The a^∗^ and b^∗^ values of the masks were calculated using the unstandardized beta values for the regression model of VO_2_ max predicting skin color (0.033 for a^∗^ and 0.076 for b^∗^, Figure 1). Image lightness was not changed as L^∗^ was not related to fitness. The average skin color of the participants (L^∗^ = 64.5, a^∗^ = 9.4, b^∗^ = 16.7) was used as a baseline with a simulated increase and decrease in VO_2_ max of 80 ml/min/kg representing High and Low mask coloration (Figure 4, middle pair L^∗^ = 64.5, a^∗^ = 12.04, b^∗^ = 22.78 and L^∗^ = 64.5, a^∗^ = 6.76, b^∗^ = 10.62, respectively). Masks were constructed to have a large color difference to minimize rounding errors. Images simulating a high fitness (VO_2_ max = 90) were produced by adding 0.25 of the color difference between High and Low masks to each composite face [Figure 4, top right see Stephen et al. (2011), Whitehead et al. (2012), Lefevre and Perrett (2015)]. Likewise, images simulating a low fitness (VO_2_ max = 10) were produced by subtracting 0.25 of the color difference between masks (Figure 4, bottom right). For each composite image, continua were produced comprised of 13 images spanning a color range of delta E = 6.63 and an associated range in VO_2_ max of 10–90 ml/min/kg (Figure 4, top and bottom right). This equates to an intended fitness change of 6.67 ml/min/kg and color change of delta E = 0.55 per image with the original face at the center of each continuum.

#### Ethics Statement

Approval from the University of St Andrews Ethics Committee was obtained prior to the study (PS12612). Participants gave informed consent and were reimbursed at a rate of £5 per hour.

#### Interactive Continua

Participants were asked to manipulate the skin color of color-calibrated Caucasian facial images along a continuum to “make the face as healthy as possible” (Stephen et al., 2011). There were four continua, one for each of the composite facial images.

#### Forced-Choice Preference

Participants then began forced-choice preference trials, choosing which of two images appeared healthier. The image pair had the same identity but varied in skin color. The image displayed from the pair was controlled by moving the mouse cursor left or right. The task began with the image pairs having a large color difference (steps of 6.67 ml/min/kg from the interactive continuum). Next the color differences ranged over three, two, and one step in random order. Each of the four composite faces was tested twice for each of the four difficulty step sizes. The actual color change between stimuli was measured by cutting a rectangular skin patch from a standard location on the forehead of each image and calculating the average L^∗^a^∗^b^∗^ values of pixels within the patches. The measured color change for the smallest step was on average delta E = 0.66.

### Perceptual Results

**FIGURE 5:**
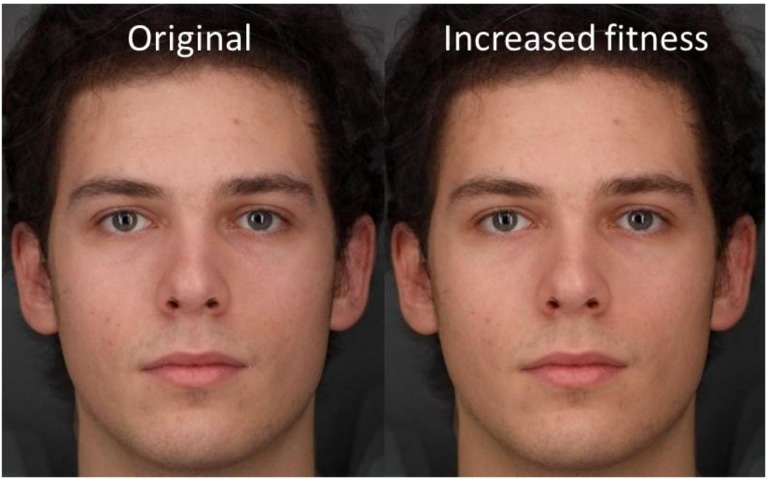
The left image represents the baseline color. The right image represents the color chosen by participants when asked to select the healthiest color from the continuum available. The change in color represents an increase in fitness level VO2 max = 23.9 ml/min/kg above baseline. Facial images are averages of three individuals and do not represent a real identity. Written informed consent was obtained from all three individuals for the publication of their image.

When making the face as healthy as possible, participants chose to increase the fitness associated color [*t*(20) = 8.463, *P* < 0.001]. On average, the skin color chosen corresponded to an increase in VO_2_ max of 23.9 ml/min/kg above the baseline original image (see Figure 5).

**FIGURE 6:**
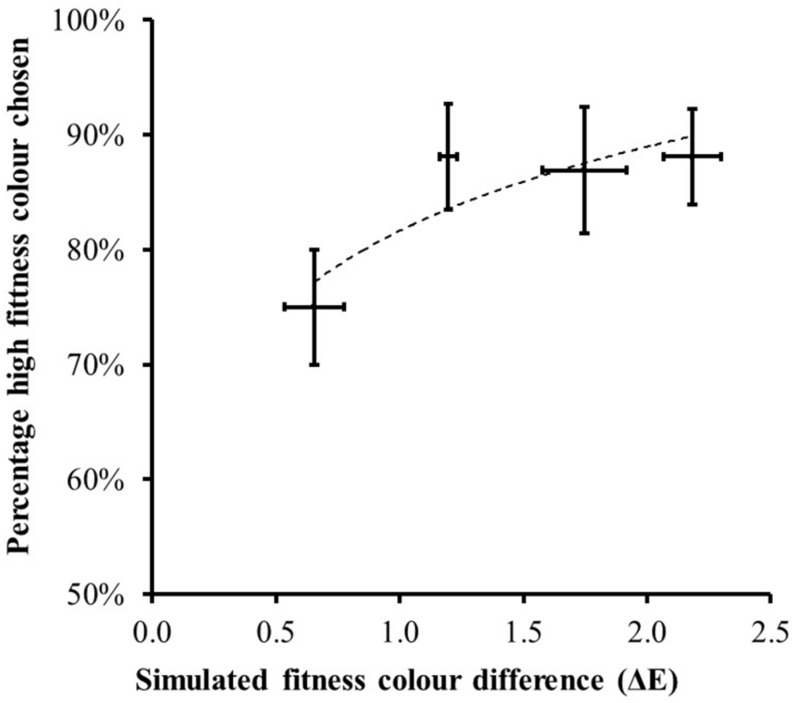
The percentage of trials (mean ± 1 standard error) where the higher fitness-associated facial color was chosen as healthiest in the forced-choice trials. Horizontal bars record the mean ± 1 standard error of the measured color difference across the eight instances of each difficult level. As the color difference between the face images increased, participants chose the fitness-associated facial coloration on a greater percentage of trials. The dotted line shows the best fit second-order polynomial.

When making a forced-choice preference, participants on average chose the facial image with increased fitness-associated color on 85% of the trials across all step sizes. This was significantly higher than chance [*t*(20) = 9.04, *P* < 0.0005]. Figure 6 illustrates that, as the color difference between the two displayed face images increased, participants chose the face with increased fitness coloration on a greater proportion of trials [*F*(1.96,39.30) = 3.895, *P* = 0.029]. Even at the smallest color difference (delta E = 0.66) the higher fitness-associated color was chosen on 75% of trials [greater than expected by chance *t*(20) = 5.12, *P* < 0.0005].

### Discussion

The results of the perceptual study show that the skin color associated with raised aerobic fitness is preferred to that associated with lowered fitness. The results also define the change in fitness required to produce a preference as < 8 ml/min/kg. We did not explore the skin tone specifically associated with raised and lowered body fat or indeed changes in sleep and stress but note that the color difference associated with these other aspects of health is similar to that associated with differences in fitness and dietary carotenoid intake, in that all affect skin yellowness but not skin lightness.

## General Discussion

This is the first demonstration that objective measures of general health (fitness and body fat%) in normal adults are related to variation in skin color. Further subjective measures (self-reported stress and habitual sleep) which are likely to be reflected in health status were also implicated in influencing skin color. While carotenoids have a health signaling role in many species, to date there has been a lack of consistent evidence that skin carotenoids are a cue to human health (Foo et al., 2017a, b; Phalane et al., 2017). Our studies suggest that multiple aspects of general health are reflected in skin carotenoids in a way that is visible to human observers as shown in study 3 (and see discussion below). Thus, our results indicate that carotenoids can function in humans as they do in other species to provide a cue to health.

As noted above, raised fitness, low body fat levels, as well as reduced stress and increased sleep were all associated with higher skin yellowness which is characteristic of raised carotenoid levels in the skin. Fitness and body fat levels were unrelated to skin lightness which would be expected if these aspects of health were related to melanin levels in the skin. Raised fitness and lowered body fat were also related to increased light absorption at wavelengths 430–520 nm which is expected to accompany raised skin carotenoid levels (Figure 2).

Multiple regression in study 1 and 2 showed that the effects of fitness and body fat levels on skin yellowness were largely independent. The effect of fitness on skin color could be mediated by an increase in efficiency of endogenous antioxidants which would protect carotenoids allowing them to accumulate in the skin. This hypothesized mechanism is consistent with our findings, although not tested directly.

We found an association between higher body fat and lower skin yellowness, supporting the hypothesized relationship between body fat and decreased carotenoid pigmentation. In studies 1 and 2, regression showed that body fat contributed to variance in skin yellowness independent of fitness, fruit and vegetable intake, and reported colds and flu symptoms. As noted, there are several routes by which body fat might have a negative impact on carotenoids, including an increase in oxidative stress. Future measurements of oxidative stress could confirm this explanation of the negative effect of body fat on skin yellowness.

In study 1, we did not find a significant relationship between fruit and vegetable consumption and skin yellowness as has been previously been reported (e.g., 11, 13). In study 2, there was a trend for the dietary–skin color association in the expected direction. The lack of clear replication may reflect the fact that we employed only a brief food frequency questionnaire. In study 1, stress and sleep were not associated with skin color, but in study 2 they were. The discrepancy may reflect the different sensitivity of longitudinal and cross-sectional assessments or it may reflect the time of year. Study 2 involved testing in September at the beginning of the university year and in November when there were course deadlines and exams. These times are likely to be associated with changes in stress and sleep patterns. Study 1 was made in early April at a time when students were without deadlines and were less likely to be making new friendships.

Fitness was related to greater skin yellowness in studies 1 and 2 and, to a lesser extent, skin redness in study 1. Figure 2 shows that fitness and body fat affect skin reflectance in a manner that parallels the light absorption of carotenoid pigments but is unlike the absorption of oxygenated or deoxygenated hemoglobin. The increase in redness with fitness in study 1 is thus likely to reflect an increase in skin carotenoids. Indeed carotenoid supplements can elevate skin redness (Stephen et al., 2011; Coetzee and Perrett, 2014).

### Limitations

Limitations of the current studies concern the limited range of ethnicity, age, and background across participants. Our participants were young adults (18–36 years old). It will require further studies to establish whether the relationships seen between skin color and aspects of a healthy lifestyle hold in older or younger populations. The current study had very few participants with dark skin. It may be that in populations with darker skin that the yellow pigmentation from carotenoids is more difficult to detect, and therefore the relation between skin yellowness and aspects of a healthy lifestyle less visible. For dark skinned individuals carotenoid skin color changes may still be visible in lightly pigmented body areas such as the palm which has a lower melanin concentration compared to other skin areas (Coetzee and Perrett, 2014). There is also cultural variation in the preference for carotenoid skin color that we find associated with fitness and health (Stephen et al., 2011; Whitehead et al., 2012; Lefevre and Perrett, 2015; Han et al., 2018; Tan and Stephen, 2019).

Our participants were all attending University which meant that they were likely to be from a relatively advantaged background. Nonetheless, variation in background deprivation may have contributed to variation in health. From psychosocial influences on well-being, we would expect deprivation to be linked to poorer diet, lower aerobic fitness, raised body fat levels, higher self-reported sickness and stress, and poorer quality sleep. This suggests that our health measures would be positively interrelated. Interrelation of health variables is also expected from the influence of personality (Burrows et al., 2015). Table 1 provides only limited evidence for such interrelations. Fitness does correlate with lower body fat and shows a trend to relate to higher fruit and vegetable consumption. Reported stress was marginally related to reported sickness but was unrelated to other health measures. Similarly, Table 3 shows little positive relation between changes in health variables. Stress increase was associated with longer rather than shorter sleep. Losing weight was associated with an increase rather than a decrease in self-reported illness. With a larger sample purposely chosen to reflect more diverse backgrounds, we might see more evidence for health variables interrelating positively.

It is appropriate to consider whether or not the effects of higher fitness and lower body fat on skin color have real life consequences for appearance. One can estimate from the regression model coefficient (Figure 1A) that a change in VO_2_ max of 8.75 ml/kg/min would be necessary for a visibly detectable change in skin color (0.7 delta E; Re et al., 2011; Whitehead et al., 2012). Study 3 showed empirically that an increase in fitness associated color equivalent to change in VO_2_ max of 7.9 ml/kg/min was preferred by observers on 75% of trials in forced-choice preference tests. Given the average VO_2_ max of 46.4 in this sample, this corresponds to a 17% change in cardiovascular fitness. Such a change is substantial but attainable given, that in 20 weeks training, the change in VO_2_ max across individuals has been seen to range from –5 to 55% (average 17%) (Bouchard et al., 1999). With similar considerations one can estimate from Figure 1B that a change in body fat of 10% would be necessary for a detectable color change. In our sample, the baseline range of body fat was 4–30% for men and 12–42% for women. Hence, change sufficient to affect color appearance is again possible.

Likewise, one can estimate that changing habitual sleep patterns to lose an average of 1.6 h of sleep per night would decrease skin yellowness perceptibly. Conversely, regaining lost sleep should re-establish the color, yet it is not clear how long the changed sleep regimen needs to persist for the color changes. The sampling interval in study 2 was 8 weeks which suggests an upper limit of 2 months but changes could be more rapid.

It is important to note that the impact of body composition, fitness, and sleep on skin color are independent of each other and independent of diet which means that improvements in different aspects of lifestyle are synergistic in instilling benefits to health and appearance. Moreover, lifestyle also impacts on body shape so appearance benefits will accrue across shape and color domains.

Motivation to exercise is important since even small improvements in fitness can have large benefits to life expectancy (Warburton et al., 2006). While the benefits of lifestyle to body shape are common knowledge, our work is the first to reveal additional benefits to skin appearance. The independent effects of dietary fruit and vegetables, fitness, and low body fat on skin color mean that multiple aspects of a healthy lifestyle each act in an additive manner to improve skin appearance. Study 2 indicates that further aspects of a healthy lifestyle, namely, avoiding stress and sleep loss, could make further contributions to improving skin color. Study 2 also shows that the skin color changes accompanying lifestyle change occur relatively quickly, within 8 weeks. It is relevant that change in intake of fruit and vegetables noticeably affects skin color within 4–6 weeks (Whitehead et al., 2012) and seeing the benefits to skin color can motivate durable changes in diet (Whitehead et al., 2014). The prospects of an attractive skin tone may help motivate people to avoid excess calorie intake, to eat more fruit and vegetables, to exercise, to reduce stress, and to follow good sleep hygiene. Such a global approach to a healthy lifestyle, rather than an exclusive focus on one variable, such as weight, fits well with health recommendations.

## Data Availability Statement

The datasets generated for this study are available on request to the corresponding author.

## Ethics Statement

The studies involving human participants were reviewed and approved by the St Andrews University Teaching and Research Ethics Committee. The patients/participants provided their written informed consent to participate in this study.

## Author Contributions

DP conceived the experiments. ST, PC, and AH conducted the experiments. DP and ST analyzed the results. All authors contributed to manuscript revision and read and approved the submitted version.

## Conflict of Interest

The authors declare that the research was conducted in the absence of any commercial or financial relationships that could be construed as a potential conflict of interest.
